# Factors related to initial treatment for adhesive capsulitis in the medicare population

**DOI:** 10.1186/s12877-022-03230-0

**Published:** 2022-06-30

**Authors:** Sarah B. Floyd, Sara M. Sarasua, Stephan G. Pill, Ellen Shanley, John M. Brooks

**Affiliations:** 1 Department of Public Health Sciences, Clemson University, Clemson, SC USA; 2 Center for Effectiveness Research in Orthopaedics, Greenville, USA; 3 School of Nursing, Clemson University, Clemson, SC USA; 4 Steadman Hawkins Clinic of the Carolinas, Prisma Health, Greenville, SC USA; 5 ATI Physical Therapy, Greenville, SC USA; 6 Department of Health Services Policy and Management, University of South Carolina, Columbia, USA

**Keywords:** Primary adhesive capsulitis, Medicare, Treatment utilization, Surgery, Physical therapy, Injections

## Abstract

**Background:**

Primary adhesive capsulitis (AC) is not well understood, and controversy remains about the most effective treatment approaches. Even less is known about the treatment of AC in the Medicare population. We aimed to fully characterize initial treatment for AC in terms of initial treatment utilization, timing of initial treatments and treatment combinations.

**Methods:**

Using United States Medicare claims from 2010–2012, we explored treatment utilization and patient characteristics associated with initial treatment for primary AC among 7,181 Medicare beneficiaries. Patients with primary AC were identified as patients seeking care for a new shoulder complaint in 2011, with the first visit related to shoulder referred to as the index date, an x-ray or MRI of the shoulder region, and two separate diagnoses of AC (ICD-9-CM codes: 726.00). The treatment period was defined as the 90 days immediately following the index shoulder visit. A multivariable logistic model was used to assess baseline patient factors associated with receiving surgery within the treatment period.

**Results:**

Ninety percent of beneficiaries with primary AC received treatment within 90 days of their index shoulder visit. Physical therapy (PT) alone (41%) and injection combined with PT (34%) were the most common treatment approaches. Similar patient profiles emerged across treatment groups, with higher proportions of racial minorities, socioeconomically disadvantaged and more frail patients favoring injections or watchful waiting. Black beneficiaries (OR = 0.37, [0.16, 0.86]) and those residing in the northeast (OR = 0.36, [0.18, 0.69]) had significantly lower odds of receiving surgery in the treatment period. Conversely, younger beneficiaries aged 66–69 years (OR = 6.75, [2.12, 21.52]) and 70–75 years (OR = 5.37, [1.67, 17.17]) and beneficiaries with type 2 diabetes had significantly higher odds of receiving surgery (OR = 1.41, [1.03, 1.92]).

**Conclusions:**

Factors such as patient baseline health and socioeconomic characteristics appear to be important for physicians and Medicare beneficiaries making treatment decisions for primary AC.

**Supplementary Information:**

The online version contains supplementary material available at 10.1186/s12877-022-03230-0.

## Background

Adhesive capsulitis (AC), often referred to as “frozen shoulder”, is a debilitating shoulder condition characterized by pain and restricted range of motion [1, 2]. AC can be classified as primary or secondary. While secondary AC more commonly occurs following shoulder trauma or shoulder surgery [3, 4], primary AC remains a mystery to clinicians and researchers and presents spontaneously without any known triggering event [1]. AC affects approximately 3–5% of the population [5], and most commonly presents in women between the ages of 40 and 60 [3, 4], although it also affects elderly adults over the age of sixty [6]. In addition to uncertainty about the etiology, controversy also remains about the most effective treatment approaches [7]. Analysis is needed to describe the range of AC treatments used in the elderly as a foundation for comparative effectiveness analysis.

Treatments for AC range from watchful waiting to surgical intervention [3, 8] with the goal of treatment to reduce pain and increase mobility and range of motion in the shoulder joint [4, 9, 10]. AC presents a unique challenge to physicians in that the presentation of symptoms and natural progression through the phases of disease varies across patients [1]. Some cases of AC improve with watchful waiting or lower levels of intervention such as injections or physical therapy (PT). Whereas other patients resort to surgical intervention. Corticosteroid injections have been shown to provide fast-acting pain relief for patients, though results are not long-lasting [4]. PT is the most common treatment for AC and has been shown to improve shoulder function and decrease pain, with the combination of PT and injections resulting in better outcomes than either one alone [4]. Additionally, due to the self-limiting nature of AC, some patients and providers may choose to neglect treating AC in favor of “watchful waiting,” allowing the disease to resolve without intervention [3].

Patients who do not respond to these conservative treatments may go on to receive more invasive treatments such as suprascapular nerve blocks (SSNB), hydrodilation, manipulation under anesthesia (MUA), arthroscopy, or open release [4]. Given the relatively low risk associated with conservative treatment options such as injections, and PT, these are likely to be the initial treatment options for most patients; however, little is known about what patient characteristics influence initial treatment choice. Though many studies have compared the efficacy of various treatment options for primary AC, no clinically superior approach has been identified [10–13], suggesting that optimal treatment choice likely varies across patients [8, 14–18]. Yet the initial treatment choice is important, with recovery taking as long as 1–4 years [19], and up to 50% of beneficiaries reporting long-lasting effects [2, 20, 21].

The objective of this analysis was to describe initial treatment utilization in a cohort of Medicare beneficiaries with a new diagnosis of primary AC. We present a descriptive summary of treatment utilization and patient characteristics associated with receiving treatment. This analysis uses complete Medicare claims data which allows for a comprehensive view of all healthcare utilization received by a nationally representative sample of beneficiaries with primary AC. Our study is one of the first to characterize Medicare patients with primary AC and describe treatment utilization for AC in the Medicare population.

## Methods

### Data and Sample

This study used United States Medicare administrative claims data from the years 2010–2012 for all fee-for-service Medicare beneficiaries with a diagnosis of any of 192 shoulder conditions in Medicare Part B claims in 2011. The comprehensive list of 192 shoulder diagnosis codes was developed with clinical co-investigators to represent all shoulder diagnoses that could occur. From this database we isolated all beneficiaries with a new diagnosis of a shoulder complaint in 2011. This served as the sampling base from which we identified patients with primary AC. The use of complete Medicare administrative data enabled patient healthcare utilization to be tracked across inpatient and outpatient settings. This study was approved by the University of South Carolina Institutional Review Board (Study Number: Pro00088703).

AC can be a difficult shoulder condition to diagnose because its clinical presentation often overlaps with other common shoulder pathology. Therefore, multiple inclusion criteria were applied to identify a cohort of new cases of primary AC from Medicare claims. First, using Medicare Part B carrier claims, the index shoulder visit was defined for each Medicare beneficiary as the first date a shoulder diagnosis was received in 2011. Next, beneficiaries with a diagnosis of AC (ICD-9-CM code: 726.00) within 90 days of the index shoulder visit and a shoulder x-ray or MRI = 30 days before or after the index shoulder visit were identified. The requirement of diagnostic imaging increased the likelihood the AC case was accurately and newly diagnosed, as an X-ray or MRI is commonly part of the differential diagnosis process [1, 20]. A second AC diagnosis within 180 days of the first AC diagnosis was required to confirm the initial AC diagnosis. Given the known association between shoulder trauma, shoulder surgery, mastectomy and secondary AC [21–23], beneficiaries with any shoulder diagnosis or claim for a mastectomy procedure in the 365-days prior to their index shoulder diagnosis were excluded from the study. Additionally, rotator cuff pathology presents similarly to AC, so any patients with a concurrent diagnosis for rotator cuff pathology were excluded.

Additional inclusion criteria applied to assure complete data for consistent measurement included (1) continuous enrollment in fee-for-service Medicare Part A and Part B from 365-days prior to 365-days after the index shoulder diagnosis and no enrollment in Medicare Part C during the study period and (2) minimum age criterion of 66 years on the index shoulder visit. The minimum age criterion of 66 years was used to ensure enrollment in the Medicare system for a year prior to the index shoulder diagnosis. The final primary AC case cohort included 7,181 Medicare beneficiaries. Supplemental Figs. 1 and 2 in the Appendix shows the patient inclusion criteria in detail.

### Treatment Measures

The treatment period represented the 90-day period beginning on the day of the index shoulder diagnosis and ending 90 days thereafter (Day 0–90). Treatment groups were created to reflect all shoulder-related treatment received during the treatment period. Treatment groups were specified as 1) PT only, 2) Injection only, 3) Injection and PT, and 3) Surgery. Beneficiaries in the surgery group may have also received PT and/or injections but due to the small size of the surgery group it was not further stratified. In order to maintain a focus on initial treatment and avoid post-operative treatment utilization, only injections and PT occurring prior to surgery were included in the analysis for surgical patients. Beneficiaries receiving no shoulder treatment during the treatment period were classified as watchful waiting (WW).

Treatment claims were identified using Part B carrier, outpatient, and Medpar Inpatient claims files. The type of treatment beneficiaries received was identified using ICD-9-CM procedure and Healthcare Common Procedure Coding System (HCPCS) codes. Treatments included: Physical therapy (HCPCS: 97,001, 97,002, 97,003, 97,004, 97,110, 97,140, 97,014, 97,112, 97,350, 97,032, 97,016, 97,124, 29,240), injection (HCPCS: 20,610, 20,611, 77,002), manipulation under anesthesia (MUA) (HCPCS: 23,700) and capsular release (HCPCS: 29,820, 29,821, 29,822, 29,823, 23,020, 29,825). Supplemental Table 1 in the Appendix contains a complete listing of procedures codes used for treatment identification. Beneficiaries receiving MUA or capsular release were considered surgically managed beneficiaries. Treatment was fully described by exploring the number of PT sessions and injections received by patients. In addition, the number of days between the index shoulder visit and AC diagnosis, and the number of days between AC diagnosis and treatment initiation was reported.

### Baseline Patient Characteristics

Patient demographic characteristics were measured by cross referencing the 2011 Beneficiary Summary Files from Medicare. Specific patient-level variables included age, sex, race, and Medicaid dual-eligibility status. General patient health was measured using Part A and B Medicare spending in the year prior to the index shoulder date [22], the Charlson Comorbidity Index (CCI), and the Frailty Risk Index (FRI). CCI is a validated measure of burden of disease [23–25]. Comorbidities are weighted from 1 to 6 for mortality risk and disease severity, and then summed to form the total CCI score. The FRI score is a validated instrument for assessing frailty among older persons [26]. Medicare Part B claims in the 365-days prior to the index shoulder date were assessed for the presence of comorbid conditions. Comorbid conditions that have been previously associated with AC were identified using ICD-9-CM codes as follows: thyroid disorder (240–246), Type 1 and Type 2 diabetes (250.XX-250.93), hyperlipidemia (272.0–272.9), hypertension (401–405), rheumatoid arthritis (714.0), gout (274), and obesity (278.X).

### Analytical Approach

Descriptive statistics summarizing patient characteristics of the AC cohort and by treatment group are presented. Differences in patient characteristics were compared across treatment groups and were assessed by ANOVA for continuous variables and chi-square tests for categorical variables. Logistic regression was used to estimate the independent influence of patient characteristics on probability of receiving surgical treatment for AC during the initial 90-day treatment period. Results are presented as adjusted Odds Ratios (OR) with accompanying 95% confidence intervals (95% CI). SAS software 9.4 (SAS Institute Inc., Cary, NC, USA) was used for data manipulation and analysis. A *P* value of < 0.05 was deemed statistically significant.

## Results

### Study Sample

In 2011, there were 7,181 Medicare beneficiaries with primary AC and a sufficient observation period to measure baseline and treatment variables. More of the sample was female (64%) than male, and the mean age of the cohort was 74.8 years. The sample was primarily white (88.7%), followed by 6.3% African American, 2.3% Other race and 1.7% Asian. Among the sample, 7.1% were dual-eligible for Medicaid on their index shoulder visit. Thirty-three percent had a CCI score of 0 and 57.5% had a FRI score of zero. Nearly one-third of the AC cohort had type 2 diabetes (29.7%), and 44.9% and 53.7% of the sample had a diagnosis of hyperlipidemia and hypertension in the previous 365 days, respectively. Sixteen percent of the sample also had a previous diagnosis of a thyroid disorder. Table 1 contains more details on the AC study sample.

**Table 1:** Patient characteristics for medicare beneficiaries with primary adhesive capsulitis cases by treatment group

	Adhesive Capsulitis Cases (*N* = 7181)	Watchful Waiting (*N* = 675)	Injection only (*N* = 901)	PT only (*N* = 2937)	Injection + PT (*N* = 2469)	Surgery (*N* = 199)	*p*-value
	N (%)	%	%	%	%	%	
*Male*	2570 (35.8)	37.8	38.3	34.4	35.6	41.2	0.09*
***Race***							< 0.01*
**Asian**	**122 (1.7)**	**2.7**	**1.9**	**1.7**	**1.5**	**0.5**	
**Black**	**452 (6.3)**	**9.0**	**7.2**	**5.9**	**6.0**	**3.0**	
**Hispanic**	**71 (1.0)**	**2.2**	**1.3**	**0.7**	**0.9**	**1.0**	
**Other**	**165 (2.3)**	**2.1**	**1.1**	**2.6**	**2.4**	**2.5**	
**White**	**6369 (88.7)**	**84.0**	**88.5**	**89.1**	**89.2**	**93.0**	
***Dual eligible for Medicaid***^a^	**509 (7.1)**	**11.9**	**11.3**	**6.2**	**5.3**	**7.0**	< 0.0001*
***Mean Age (SD)***	**74.8 (6.8)**	**76.3 (7.4)**	**76.2 (7.1)**	**74.9 (6.9)**	**74.0 (6.4)**	**71.7 (5.1)**	** < 0.0001**^**†**^
***Age Group***							< 0.0001*
**66–69**	**2262 (31.5)**	**26.5**	**24.1**	**31.3**	**34.7**	**47.2**	
**70–75**	**2297 (32)**	**28.7**	**29.5**	**31.8**	**33.6**	**36.2**	
**76–79**	**998 (13.9)**	**13.8**	**17.8**	**13.8**	**13.1**	**8.0**	
**80–85**	**1055 (14.7)**	**19.0**	**18.4**	**14.8**	**12.5**	**7.0**	
**86 + **	**574 (8.0)**	**11.9**	**10.2**	**8.4**	**6.0**	**1.5**	
***Region***							< 0.0001*
**Midwest**	**1644 (22.9)**	**21.9**	**16.8**	**23.4**	**24.3**	**27.1**	
**Northeast**	**1450 (20.2)**	**21.6**	**19.5**	**22.3**	**18.6**	**6.5**	
**South**	**2958 (41.2)**	**37.3**	**48.8**	**37.0**	**43.5**	**51.3**	
**West**	**1134 (15.8)**	**19.1**	**14.9**	**17.2**	**13.6**	**15.1**	
***Charlson Comorbidity Index (CCI)***							< 0.001*
**0**	**2355 (32.8)**	**29.3**	**27.5**	**34.1**	**34.2**	**32.2**	
**1**	**1572 (21.9)**	**19.4**	**21.1**	**21.8**	**22.6**	**26.6**	
**2**	**1041 (14.5)**	**14.4**	**15.9**	**15.0**	**13.5**	**13.1**	
**3**	**876 (12.2)**	**14.2**	**13.2**	**11.9**	**11.7**	**12.1**	
**4 + **	**1335 (18.6)**	**22.7**	**22.3**	**17.3**	**18.0**	**16.1**	
***Frailty Risk Index (FRI)***							< 0.0001*
**0**	**4129 (57.5)**	**49.9**	**53.5**	**60.2**	**57.5**	**64.8**	
**1**	**1809 (25.2)**	**27.1**	**24.1**	**24.0**	**26.6**	**24.1**	
**2**	**675 (9.4)**	**10.1**	**9.5**	**9.0**	**9.9**	**5.5**	
**3 + **	**567 (7.9)**	**12.9**	**12.9**	**7.0**	**6.0**	**5.5**	
*Comorbidities*							
Type 1 Diabetes	244 (3.4)	3.3	4.6	3.2	3.2	2.5	0.17*
**Type 2 Diabetes**	**2132 (29.7)**	**30.4**	**30.6**	**28.4**	**30.2**	**35.2**	0.05*
Hyperlipidemia	3224 (44.9)	41.5	44.3	44.1	47.0	44.7	0.08*
**Hypertension**	**3856 (53.7)**	**56.4**	**57.2**	**52.8**	**53.3**	**47.2**	0.02*
Thyroid Disorder	1170 (16.3)	16.9	18.8	15.7	16.3	12.1	0.12*
**Rheumatoid arthritis**	**129 (1.8)**	**2.7**	**3.8**	**1.4**	**1.5**	**0.5**	< 0.0001*
Gout	179 (2.5)	3.0	3.3	2.1	2.5	2.0	0.27*
Parkinson’s Disease	93 (1.3)	0.9	1.3	1.4	1.3	0.5	0.88*
Obesity	57 (0.8)	0.6	0.8	0.8	0.9	2.0	0.24*
***Mean Previous Year Medicare Spending (SD)***^b^	**$10,183 (20,995)**	**$12,403 (24,546)**	**$13,875 (27,669)**	**$9737** **(21,440)**	**$8967 (16,201)**	**$7613 (12,064)**	** < 0.0001**^**†**^

*Abbreviations*: *PT* Physical Therapy, *SD* Standard Deviation

Statistically significant results (*p* < 0.05) in bold

^*^Chi-Square Test. ^**†**^ANOVA for continuous variables

^a^Beneficiary was fully dual-eligible for Medicare and Medicaid during the month the index shoulder visit occurred

^b^Total Part A and B payments made by Medicare for the beneficiary over the period of 365-days prior to their index shoulder date

Over ninety percent of the sample received at least one treatment (PT, injection, or surgery) in the treatment period. PT alone was the most common initial treatment, with 41% of the AC cohort receiving this treatment modality in the initial 90-day treatment period. Injection combined with PT was the second most common treatment approach with 34% of the sample receiving these two treatments during the treatment period. Injection alone was used by 901 beneficiaries or 12.5% of the AC sample. Surgical intervention within the first 90 days was used by 199 beneficiaries or 2.7% of the AC cohort.

### Patient Characteristics and Initial Treatment

Some similar trends emerged in patient characteristics or patient profiles across treatment groups. Three distinct patient profiles that seemed to follow baseline patient health and socioeconomic characteristics emerged. Patients in the 1) WW and injection treatment groups, 2) the PT alone and Injections and PT groups, and 3) the surgery group represented distinct patient profiles. Based on age, race, dual-eligibility, CCI and FRI scores, comorbidity prevalence and previous Medicare spending, patients in the WW and injection groups appeared to be in the poorest health, patients in the PT and Injections and PT treatment groups appeared to be in middle health and then patients receiving surgery were in the best health and socioeconomic standing.

The WW and injection treatment groups had similar patient characteristics in terms of race, age, dual-eligibility, CCI and FRI scores, prevalence of hypertension and previous Medicare spending. The WW and injection groups had the highest proportion of all racial minorities (Asian, Black, Hispanic and Other race) with 16% and 11.5% of patients in each respective treatment group being a racial minority. These two treatment groups also had the oldest beneficiaries with the average age of beneficiaries being 76.3 and 76.2 years of age. Both the WW and injection groups also had the highest proportion of dual-eligible beneficiaries with 11.9% and 11.3% being dual-eligible for Medicaid. Additionally, patients in the WW and injection groups had the highest levels of previous Medicare spending ($12,403 and $13,875, respectively).

Patients receiving PT alone or Injections and PT also appeared more similar. The PT and Injections and PT treatment groups had similar patient characteristics in terms of race, age, dual-eligibility, CCI and FRI scores. The age of patients in PT only and Injections and PT groups was similar and on average was 74.9 and 74.0 years of age. The PT only and Injections and PT treatment groups had the lowest proportion of Medicaid dual-eligible beneficiaries at 6.2% and 5.3%, respectively. Patients in these two groups had similar levels of previous Medicare spending of $9,737 and $8,967, respectively.

Patients receiving surgery were on average the youngest and had the lowest FRI scores. A larger proportion of beneficiaries in the surgery treatment group had a FRI score of 0 (64.8%), compared to other treatment groups. The surgery treatment group had the highest proportion of white beneficiaries (93.0%) and beneficiaries in the surgery group were the youngest with the average age of 71.7 years. Regional variation in treatment utilization was observed with the surgery treatment group having the highest proportion of beneficiaries residing in the south (51.3%). A significantly higher proportion of beneficiaries with type 2 diabetes received initial surgery (35.2%) and a significantly lower proportion of beneficiaries with hypertension (47.2%) and rheumatoid arthritis (0.5%) received initial surgery. Beneficiaries receiving initial surgery also had significantly lower Medicare spending in the 365 days prior to their index shoulder visit ($7,613) compared to patients in the other treatment groups. Table 1 contains details on the study sample and patient characteristics across initial treatment groups.

### Treatment Utilization

On average it took 18.3 days from their initial visit for a shoulder condition for beneficiaries to receive a diagnosis of AC. Those beneficiaries receiving only injections had the shortest time to AC diagnosis of only 14.8 days, whereas those beneficiaries undergoing surgery had the longest diagnosis time of 26.9 days. On average, in the complete AC cohort, treatment was initiated within 17.7 days. Treatment was initiated the fastest for the beneficiaries receiving a combination of PT and injections, with the average time to treatment being 10.4 days.

Beneficiaries in the PT only treatment group on average received 11.1 PT sessions during the 90-day treatment period. Those beneficiaries getting a combination of injections and PT received slightly less PT sessions, on average receiving 10.6 sessions. For those beneficiaries receiving only injections, the average number of injections was 1.4 during the treatment period. Beneficiaries receiving injections and PT received slightly less injections with the average number of injections being 1.24 during the treatment period. For those beneficiaries undergoing surgery, the average time to surgery was 51.0 days. Of the patients receiving surgery, 78% received either PT or injections prior to undergoing surgery. Surgery recipients completed on average 4.3 PT sessions and 0.85 injections prior to surgery during the 90-day treatment period. Full details on treatment utilization can be found in Table 2.

**Table 2:** Treatment utilization for medicare beneficiaries with primary adhesive capsulitis by treatment group

Treatment Utilization	Adhesive Capsulitis Cases (*N* = 7181)	Injection only (*N* = 901)	PT only (*N* = 2937)	Injection + PT (*N* = 2469)	Surgery (*N* = 199)	*p*-value
	*Mean*	*Mean*	*Mean*	*Mean*	*Mean*	
**Days from index shoulder visit to AC diagnosis**	**18.3**	**14.8**	**20.6**	**17.2**	**26.9**	** < 0.0001**^**†**^
**Days from index shoulder visit to first treatment**	**17.7**	**17.4**	**22.7**	**10.4**	**19.9**	** < 0.0001**^**†**^
**Number of injections over treatment period**	**1.3**	**1.4**	**-**	**1.24**	0.85*	** < 0.0001**^**†**^
**Number of PT sessions over treatment period**	**10.9**	**-**	**11.1**	**10.6**	4.3*	** < 0.0001**^**†**^
Days from index shoulder visit to surgery	51.0	-	-	-	51.0	^**†**^

*Abbreviations*: *AC* Adhesive Capsulitis, *PT* Physical Therapy

Statistically significant results (*p* < 0.05) in bold

^**†**^ANOVA for continuous variables

^*^Only PT and injections occurring prior to surgery were included in the analysis

### Patient Characteristics and Surgical Treatment

Table 3 shows results from a logistic regression model assessing patient characteristics associated with receiving surgical treatment in the 90 days following the index shoulder visit. The adjusted odds of surgery were significantly lower for beneficiaries residing in the northeast (OR = 0.36, [0.18, 0.69]) compared to beneficiaries residing in the west, and for black beneficiaries (OR = 0.37, [0.16, 0.86]) compared to white beneficiaries. On the other hand, younger beneficiaries aged 66–69 years (OR = 6.75, [2.12, 21.52]) and 70–75 years (OR = 5.37, [1.67, 17.17]) and beneficiaries with type 2 diabetes had significantly higher odds of receiving surgery (OR = 1.41, [1.03, 1.92]). No differences in odds of receiving surgery were observed by sex, frailty or dual-eligibility status.

**Table 3:** Logistic regression results for the association between patient characteristics and surgical treatment

	Logistic Regression Model
	OR of Surgery
*Sex*	
Female	*Reference*
Male	1.14 [0.84, 1.53]
*Race*	
Asian	0.23 [0.03, 1.75]
**Black**	**0.37 [0.16, 0.86]**
Hispanic	0.77 [0.18, 3.35]
Other	0.93 [0.37, 2.31]
White	*Reference*
*Dual eligible for Medicaid*^a^	1.54 [0.85, 2.78]
*Age Group*	
**66–69**	**6.75 [2.12, 21.52]**
**70–75**	**5.37 [1.68, 17.17]**
76–79	2.79 [0.81, 9.66]
80–85	2.45 [0.70, 8.58]
86 +	*Reference*
*Region*	
Midwest	1.23 [0.78, 1.95]
**Northeast**	**0.36 [0.18, 0.69]**
South	1.31 [0.86, 2.01]
West	*Reference*
*Frailty Risk Index (FRI)*	
0	1.25 [0.66, 2.37]
1	1.11 [0.57, 2.18]
2	0.79 [0.34, 1.87]
3 +	*Reference*
*Comorbidities*	
Type 1 Diabetes	0.61 [0.24, 1.54]
**Type 2 Diabetes**	**1.41 [1.03, 1.92]**
Hyperlipidemia	0.98 [0.74, 1.32]
Hypertension	0.85 [0.63, 1.14]
Thyroid Disorder	0.74 [0.47, 1.14]
Rheumatoid arthritis	0.34 [0.05, 2.46]
Gout	0.91 [0.33, 2.52]
Parkinson’s Disease	0.45 [0.06, 3.28]
Obesity	2.38 [0.83, 6.81]

The model was adjusted for all listed covariates. Statistically significant results (*p* < 0.05) in bold

^a^Beneficiary was fully dual-eligible for Medicare and Medicaid during the month the index shoulder visit occurred

Reference Groups: Non-dual eligible for Medicaid, absence of each comorbidity (Type 1 Diabetes, Type 2 Diabetes, Hyperlipidemia, Hypertension, Thyroid Disorder, Rheumatoid Arthritis, Gout, Parkinson’s Disease and Non-Obese)

## Discussion

This study is one of the first analyses describing Medicare beneficiaries with primary AC, and treatment utilization and patient characteristics associated with receiving treatment for primary AC in the Medicare population. We found variation in the initial treatments used for AC. Observed treatment variation among Medicare beneficiaries with AC reflects the lack of consensus regarding the most appropriate management of primary AC and underscores the need for better treatment effectiveness evidence.

This is one of the first analyses to describe Medicare beneficiaries diagnosed with primary AC. We found that the sample was primarily female (64%) and white (88.7%) and the average age was 74.8 years. Elderly Medicare beneficiaries tend to have more chronic conditions and a higher rate of comorbidities than the younger AC population [27], which may influence their treatment course. Over half of the cohort had a diagnosis of hypertension (53.7%) and 44.9% also had a diagnosis of hyperlipidemia, which is similar to the prevalence reported in the general fee-for-service Medicare population [27, 28]. Nearly one-third of the AC cohort had Type 2 diabetes (29.7%). Zreik, et al. (2016) found a similar prevalence of diabetes among a population of patients with AC [29].

Most of the beneficiaries with AC (> 90%) initiated shoulder treatment within the first 90 days after a shoulder diagnosis. Patient characteristics including race, age, dual-eligibility for Medicaid and measures of comorbidity, frailty and baseline health significantly differed across initial treatment groups with some similar patient profiles emerging. The patient profiles were similar between WW and injection groups. Both the WW and injection only groups had the highest proportions of racial minorities, had the oldest beneficiaries, had the highest proportions of dual-eligible beneficiaries and had beneficiaries in the poorest baseline health based on CCI Scores, FRI Scores and previous Medicare spending. The PT only and injections and PT groups appeared more similar in terms of racial makeup, age, dual-eligibility, and baseline health. The surgery group appeared unlike the conservative treatment groups and tended to be composed of younger, more white beneficiaries, and beneficiaries in better baseline health.

Beneficiaries receiving no treatment intervention (WW) or injections, were frailer and in poorer health compared to the other treatment groups. Next to WW, injections require the least amount of physical intervention or manipulation. Therefore, we hypothesize that patients that are frailer and in poorer health may choose, be guided, or engage in shared decision-making with their provider to receive WW or injections. The physical manipulation and associated shoulder pain that is customary with PT may not be feasible or desirable for this patient group in poorer health. Additionally, the finding that minority race beneficiaries and those dually-eligible for Medicaid were most likely to receive WW or injections only may reflect baseline health disparities and health care access barriers. One explanation is that beneficiaries of Black or Hispanic race and those dually-eligible for Medicaid are more likely to be in poorer health compared to white and non-eligible beneficiaries [30, 31], thus being less willing or able to perform PT. These groups are also more likely to have difficulties paying for out-of-pocket medical expenses [30, 31] and finding reliable transportation [32], both of which would limit an individual’s ability and willingness to seek injection therapy or attend regular PT sessions. Overall, there is considerable overlap in factors relevant to beneficiaries treated with WW or injections only that may have influenced this treatment choice. It appears that even among conservative management approaches (PT and injection), initial treatment choice for AC may be based on patient factors beyond clinical shoulder characteristics and may include a holistic assessment of patient baseline health, socioeconomic factors, and treatment time and pain tradeoffs that patients must consider when seeking care, highlighting the necessity of shared decision-making when treating patients with AC.

Time to initial treatment suggests that treatment for AC is initiated early and on average begins within a few weeks of an index shoulder diagnosis. To date little is known about optimal timing or progression of treatment for AC, but PT is widely accepted as an early method to initiate mobilization of the shoulder [26]. We found that PT alone or injections plus PT were the most common first line of treatment used among Medicare beneficiaries. These findings are consistent with those of Cogan et al., who found that most therapeutic intervention took place in the period 3 months before to 3 months after AC diagnosis, with opioids, physical therapy, and injections being the most common treatment approaches [33]. The effectiveness of PT during early stages of AC may be limited as pain may prohibit effective physical manipulation and strengthening exercises which are characteristic of PT intervention. Intra-articular injections have been found to reduce fibromatosis and myofibroblasts in adhesive shoulders [34] and studies support that injection provides rapid pain relief [35–38]. Therefore, there seems to be an advantage of initiating injection and PT simultaneously or in succession [39, 40].

Similar to studies within the younger AC population [41], we found that beneficiaries in the Medicare population are most often initially treated conservatively [33, 42]. The indications and optimal timing for surgical intervention for AC remain unknown, though surgical intervention is generally accepted after a period of 3 to 6 months of failed conservative treatment [7]. However, in those who underwent surgery in our sample, surgery occurred on average within 50 days. Additionally, beneficiaries receiving surgery in our cohort, also received injections and PT. On average surgery recipients completed 4 PT and 0.85 injections prior to surgery. It remains unclear why this group of patients failed both PT and injections and ultimately received surgery. The higher, focused utilization of multi-modal treatments in this group may reflect more severe or complex clinical presentation of AC. Future studies should work to identify those patients best suited for surgery and initiate surgical treatment earlier in the care seeking pathway.

We found that beneficiaries who were black or lived in the northeast had lower odds of being treated surgically within the first 90 days after a shoulder diagnosis. Racial disparities in surgery utilization have been observed for other orthopedics conditions, including knee arthroplasty [43]. Patient’s preferences for surgical treatment may differ across patients, but patients should have equal access to all available treatment options. Similarly, geographic variation and treatment signatures exist across a number of orthopaedic conditions [44, 45] and demonstrate the need for better treatment effectiveness evidence.

The association between diabetes and surgical treatment may reflect higher rates of failed conservative treatment in patients with diabetes [34]; however we do not have enough evidence to investigate this assumption and the literature on this topic yields inconsistent conclusions. Kington et al. did not find a correlation with diabetes and likelihood of surgery in a study including 2,190 beneficiaries over a 10-year period at their institution [42]. Alternatively, in a large, nationwide sample of 165,937 patients, Cogan et al. found that diabetes was associated with decreased odds of receiving surgery [33]. Therefore, more work is needed to identify the most appropriate initial treatment choice for patients with diabetes.

Effective treatment for AC remains elusive and controversial and even less is known about optimal treatment in the Medicare population. Thus, our analysis aims to describe AC treatment utilization in the Medicare population as an initial step toward needed comparative effectiveness research. There are several limitations to this study. Though Medicare data represents a large and highly generalizable population, these results are dependent on the accuracy of the diagnosis and treatment codes reported. Additionally, there may be underrepresentation of some treatment options. Our analysis did not include Part D claims and thus we did not include pharmacologic treatments in our analysis. For example, the use of nonsteroidal anti-inflammatory drugs (NSAIDs) and oral corticosteroids are excluded from our analysis. Although because NSAIDs are readily available over the counter, it is unlikely that we would have observed their utilization in medical claims data. Similarly, home exercise regimens are often used for beneficiaries with AC, so the use of PT may be underrepresented in our analysis. The ordering of multi-modal treatments was not assessed. We identified if multiple treatment modalities were used within the 90-day treatment period (i.e., injections and PT), but it is unclear if those modalities were used during the same time period or occurred in a sequential fashion. This is an important area of research as it is important to know if multiple treatments have interactive effects. We identified three patient profiles that corresponded to treatment utilization. We cannot say with certainty that AC presentation or complexity did not also correlate with the observed patient characteristics we reported, but we instead posit that other patient characteristics such as baseline health and socioeconomics is what drove the treatment choice.

In summary, the treatment of AC in the elderly population seems to follow similar treatment trends as observed in the younger population with most beneficiaries receiving initial conservative care. Though in our elderly sample, similar patient profiles emerged across treatment groups. It appears that initial treatment choice for AC may be guided by patient factors beyond clinical shoulder characteristics in the elderly population. Factors such as patient baseline health and socioeconomic factors that influence treatment choice are important for Medicare beneficiaries making treatment decisions for AC. There is a group of patients that were converted from conservative management to surgery within 90 days. At this time, it is not clear which patients are best suited for surgery, but future work should work to identify and intervene on that group early in the treatment process.

## Supplementary Information

**Additional file 1:** **Supplemental Figure 1.** Adhesive Capsulitis Inclusion Criteria Flow Chart. **Supplemental Figure 2.** Adhesive Capsulitis Cohort Identification. **Supplemental Table 1.** ICD-9 and HCPCS Codes Used for Study Identification and Exclusion Criteria.

## Data Availability

The data that support the findings of this study are available from the Center for Medicare and Medicaid Services but restrictions apply to the availability of these data, which were used under license for the current study, and so are not publicly available. Data are however available from the authors upon reasonable request and with permission of the Center for Medicare and Medicaid Services.

## Abbreviations

AC: Adhesive Capsulitis
PT: Physical Therapy
MRI: Magnetic Resonance Imaging
WW: Watchful Waiting
HCPCS: Healthcare Common Procedure Coding System
MUA: Manipulation under anesthesia
CCI: Charlson Comorbidity Index
FRI: Frailty Risk Index
SD: Standard Deviation
OR: Odds Ratio
NSAIDs: Nonsteroidal Anti-Inflammatory Drugs
