# Supplementary material for: Factors related to initial treatment for adhesive capsulitis in the medicare population
Source: BMC Geriatr. 2022 Jun 30;22:548. doi: 10.1186/s12877-022-03230-0 (PMC9248121; doi:10.1186/s12877-022-03230-0)
Supplement: Supplementary file 1 — Additional file 1: Supplemental Figure 1. Adhesive Capsulitis Inclusion Criteria Flow Chart. Supplemental Figure 2. Adhesive Capsulitis Cohort Identification. Supplemental Table 1. ICD-9 and HCPCS Codes Used for Study Identification and Exclusion Criteria. [file 12877_2022_3230_MOESM1_ESM.docx]

Appendix

Supplemental Figure 1. Adhesive Capsulitis Inclusion Criteria Flow Chart
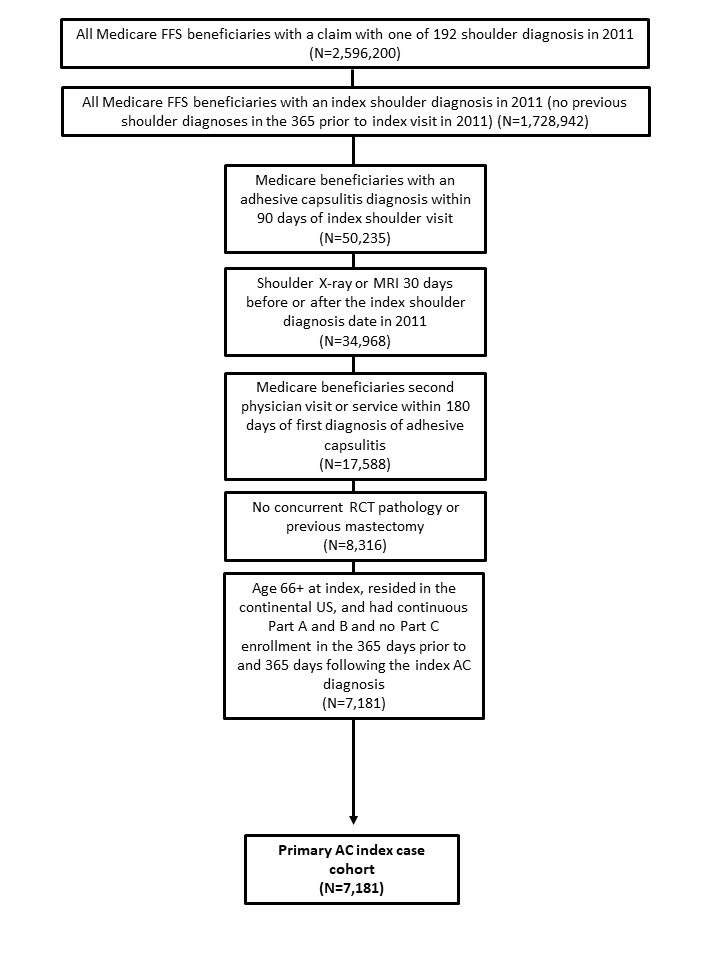


Supplemental Figure 2. Adhesive Capsulitis Cohort Identification


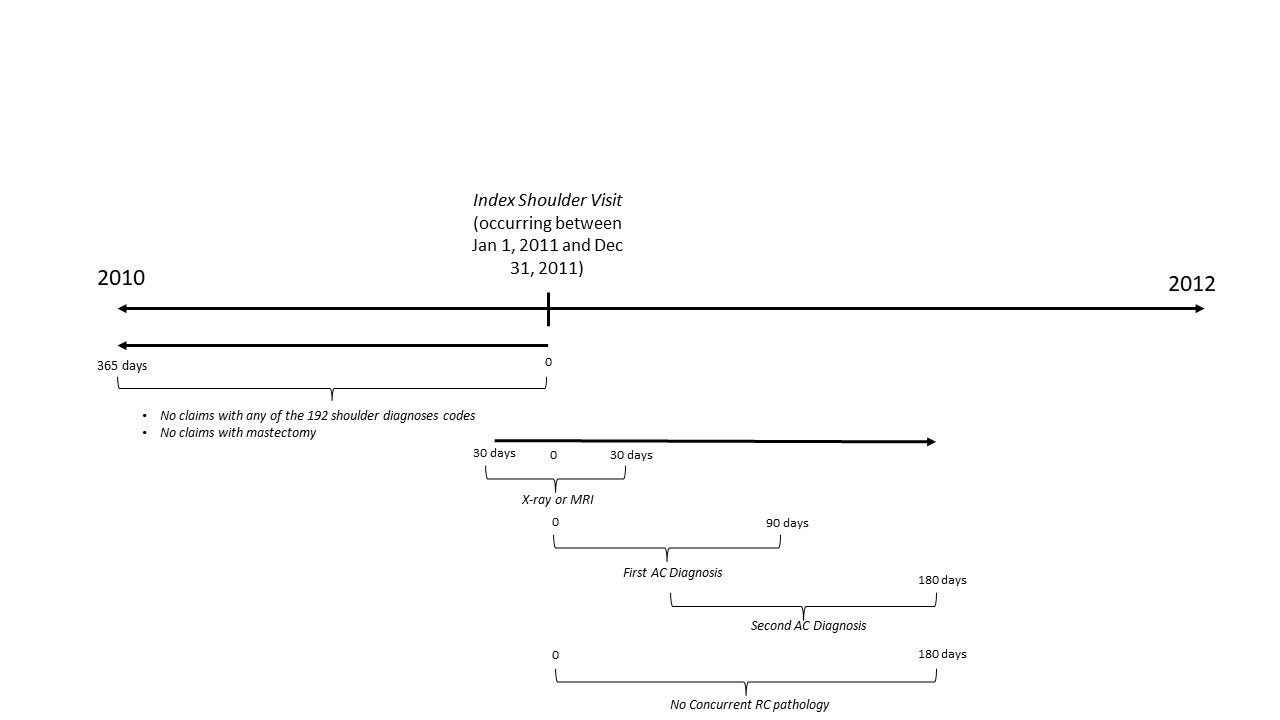


Supplemental Table 1. ICD-9 and HCPCS Codes Used for Study Identification and Exclusion Criteria

| *Shoulder Condition* | ICD-9 Diagnosis Codes |
| --- | --- |
| Adhesive Capsulitis Diagnosis | 726.00 |
| Rotator Cuff Pathology | 727.61, 840.3, 840.4, 726.10 |
| *Procedure* | HCPCS Codes |
| Mastectomy | 19160, 19162, 19180, 19182, 19200, 19220, 19240, 19301-19307 |
| Shoulder and neck region X-Ray | 73000, 73010, 73020, 73030, 73050, 73060 |
| Shoulder and neck region MRI | 73221-73223, 70540, 70542, 70543, 73218-73220 |
| *AC Treatment Modalities* |  |
| Physical Therapy | 97001, 97002, 97003, 97004, 97110, 97140, 97014, 97112, 97530, 97032, 97016, 97124, 29240 |
| Injection | 20610, 20611, 77002 |
| Capsular Release | 23020, 29825, 29820, 29821, 29822, 29823 |
| Manipulation Under Anesthesia (MUA) | 23700 |
